# Sex-related differences in care and prognosis in acute coronary syndrome

**DOI:** 10.1016/j.pmedr.2025.103131

**Published:** 2025-06-07

**Authors:** Csaba Sári, Christian M. Heesch, Attila János Kovács, Péter Andréka

**Affiliations:** a Gottsegen György National Cardiovascular Center, Budapest, Hungary; b Doctoral College of Semmelweis University, Semmelweis University, Budapest, Hungary

**Keywords:** Cardiovascular prevention, Acute coronary syndrome, Sex differences, Risk factors, Patient education

## Abstract

**Objective:**

This study investigates differences based on sex in prognosis for acute coronary syndrome using data of 76,153 patients in Hungary's National Myocardial Infarction Register, focusing on all-cause mortality, risk factors, and treatment outcomes.

**Methods:**

The data set analyzed is the result of mandatory reporting on patients presenting with acute coronary syndrome to hospitals in Hungary. The current study comprises a retrospective analysis of all cases reported to the registry from 2014 to 2019. The primary outcome measured was all-cause mortality, with an average follow-up of 3.6 years.

**Results:**

The patient cohort had a median age of 67.4 years; females were on average seven years older. Women had a less favorable cardiovascular risk profile, obtained less optimal treatment, experienced more complications, and were less likely to receive guideline-consistent therapy. An age-adjusted analysis demonstrated that age significantly influences risk profiles, types of acute coronary syndrome, treatment strategies, and outcomes. The unadjusted mortality rate for females was significantly higher; however, multivariable analysis indicated that a worse short-term prognosis was contrasted by a better long-term prognosis when comparing females to males.

**Conclusions:**

This study highlights the need to further understand and address sex differences in the presentation and management of acute coronary syndrome. The noted higher mortality rates in female patients reveal the necessity for tailored, age-adjusted treatment strategies. Women often present with more advanced cardiovascular risk profiles and face barriers to timely care, making equitable access essential. Understanding the interplay between age, sex, and comorbidities is key to improving patient outcomes.

## Highlights

• Sex differences in acute coronary syndrome patients were examined retrospectively.
• The analysis involved 76,153 patients over a five-year period.
• Women were significantly older and had a less favorable cardiovascular risk profile.
• Women faced higher rates of complications and received less optimal treatment.
• The study highlights the significance of addressing sex differences.

## 1 Objective

Cardiovascular disease is the most common cause of death in Western developed nations (Timmis et al., 2020). While cardiovascular mortality has declined in the last decades, this positive change affects women less than men (Gupta et al., 2014). In fact, women face worse outcomes in acute coronary syndrome (ACS) compared to men, including higher mortality rates (Martin et al., 2025).

Despite these sex-related discrepancies in outcomes and prognosis, research aimed at identifying the underlying causes is limited, and women are underrepresented in many clinical trials, including those related to applications for U.S. Food and Drug Administration drug approval in certain cardiovascular conditions (Scott et al., 2018). However, in trials focusing on older patient populations, such a difference is not observed (Kunadian et al., 2024). Females presenting with ACS are usually older and have more comorbidities, including hypertension, dyslipidemia, and diabetes mellitus (Hao et al., 2019; Huber et al., 2022). Further, women often present with atypical or noncardiac chest pain, or they attribute their symptoms to other conditions such as reflux disease (Lichtman et al., 2018), leading to an initial misdiagnosis or the offering of lower levels of care. Female patients themselves take longer to seek medical help after the onset of symptoms, and their electrocardiogram changes and troponin patterns often present a more difficult diagnostic challenge (Canto et al., 2007; Gebhard et al., 2019; Meyer et al., 2019; Roswell et al., 2017; Mehta et al., 2016; Lunova et al., 2023). Lastly, females are more likely to present with non-ST-segment myocardial infarction (NSTEMI), myocardial infarction with non-obstructive coronary arteries (MINOCA), spontaneous coronary dissection, coronary spasm (Smilowitz et al., 2017; Akhter et al., 2009; Thompson et al., 2005; Selzer et al., 1976), plaque erosion, and coronary thrombosis (Farb et al., 1996), all factors well known to complicate diagnosis, delay ultimate choice of treatment, and influence outcomes.

Using smaller data sets, other investigators have suggested that women are less likely to receive timely percutaneous revascularization (Du et al., 2017; Khan et al., 2018; Hao et al., 2015; Kuehnemund et al., 2021). Percutaneous coronary intervention (PCI)-related bleeding complications, cardiogenic shock, and the development of mechanical complications are more common in female patients. Lastly, women tend to have a higher Killip stage on admission (Dey et al., 2009; Berthillot et al., 2010).

During and after female patients' hospitalization, the use of guideline-directed choice of secondary prevention drugs is lower (Redfors et al., 2015; Byrne et al., 2023; Lawton et al., 2022), as is the referral to, and participation in cardiac rehabilitation programs (Resurrección et al., 2019).

## 2 Methods

### 2.1 Data source

In Hungary, as of January 2014, data on all patients who present with ACS are collected and entered into the National Myocardial Infarction Register (mandatory by legislation). As of September 2022, data on 155,000 encounters involving more than 130,000 patients had been collected. This retrospective analysis was performed based on the data from the Register. Our research was conducted using a publicly accessible, strictly anonymized database, thereby exempting it from the requirement of ethical compliance. The study complied with the institution's guidelines (Local Ethical Committee – Gottsegen National Cardiovascular Center) for protecting human subjects, prioritizing their safety and privacy throughout the research process.

### 2.2 Study population and analytical cohort

We examined 76,153 cases entered into the register from 2014 to 2019. The average duration of follow-up was 3.6 years (standard deviation: 2.4 years) as of March 25, 2022. Sex was defined as the classification associated with the binary categories of sex assigned at birth.

### 2.3 Primary outcome, statistical analysis

The primary outcome of this study was all-cause mortality, with survival time calculated in days from the initial diagnosis of myocardial infarction. The specific causes of mortality and the incidence of major adverse cardiovascular events were not identified. Baseline characteristics were summarized as medians with interquartile ranges for continuous variables and as counts with percentages for categorical variables. Baseline characteristics were assessed using the Mann-Whitney U (MWU) test for continuous variables and the chi-square (χ^2^) test for categorical variables. To evaluate differences in all-cause mortality, we conducted correlation tests followed by binary logistic regression, Cox regression, Kaplan-Meier curves, and the log-rank test, using a significance threshold of *p*-value ≤0.05. All statistical analyses were performed using SPSS Statistics, version 29.0.2.0 (IBM Corp.).

## 3 Results

A comprehensive analysis was conducted involving 76,153 patients. The cohort's median age was 67.4 years, with females accounting for 40 % of the population and being, on average, seven years older than males at the time of admission. The overall patient population showed mortality rates of 12.1 % in the short term, 21.5 % in the mid-term, and 37 % in the long term, corresponding to 30-day, 1-year, and 5-year time points after presentation. The baseline characteristics are detailed in Table 1, while the adjusted determinants of mortality assessed through multivariable statistical analysis are outlined in Table 2.

**Table 1:** Comparison of baseline characteristics of patients presenting with acute coronary syndrome in Hungary (2014–2019). All-cause mortality was assessed as of March 25,2022, with an average follow-up period of 3.6 years.

	all patients, number of cases (%), or median and IQR	females, number of cases (%), or median and IQR	males, number of cases (%), or median and IQR	p-value (chi2, *t*-test, MWU)
Sex distribution	76,153	30,552 (41.1)	45,601 (59.9)	<0.01

**Risk factors**				
age (years)	67.4 (58.5–76.8)	72.3 (62.7–80.6)	65 (56.1–73.4)	<0.01
diabetes mellitus	25,783 (33.9)	11,083 (36.3)	14,700 (32.2)	<0.01
hypertension	60,447 (79.4)	25,676 (84)	34,771 (76.3)	<0.01
hyperlipidaemia	22,647 (29.7)	9040 (29.6)	13,607 (29.8)	<0.01
anamnestic myocardial infarction	13,614 (17.9)	4861 (15.9)	8753 (19.2)	<0.01
anamnestic CABG	3578 (4.7)	1039 (3.4)	2539 (5.6)	<0.01
anamnestic PCI	11,811 (15.5)	3979 (13)	7832 (17.2)	<0.01
active smoking	19,581 (25.7)	5796 (19)	13,785 (30.2)	<0.01

**Admission**				
prehospital CPR	2513 (3.3)	839 (2.7)	1674 (3.7)	<0.01
atrial fibrillation on admission	6780 (8.9)	3150 (10.3)	3630 (8)	<0.01
Killip I on admission	62,959 (82.7)	24,429 (80)	38,530 (84.5)	<0.01
Killip II on admission	8674 (11.4)	4076 (13.3)	4598 (10.1)	<0.01
Killip III on admission	(2622) (3.4)	1183 (3.9)	1439 (3.2)	<0.01
Killip IV on admission	1162 (1.5)	517 (1.7)	645 (1.4)	<0.01
GFR on admission (ml/min/1.73 m^2^)	71 (49–89)	62 (41–82)	76 (55–92)	<0.01
serum creatinine on admission (umol/l)	82 (64–107)	76 (58–103)	86 (69–109)	<0.01

**ACS characteristics, treatment**				
STEMI diagnosed	33,843 (44.4)	12,948 (42.4)	20,895 (45.8)	<0.01
NSTEMI diagnosed	42,310 (55.6)	17,604 (57.6)	24,706 (54.6)	<0.01
PCI – during the index event	52,316 (68.7)	19,091 (62.5)	33,225 (72.9)	<0.01
direct admission to catheter centre	58,830 (77.3)	22,333 (73.1)	36,497 (80)	<0.01
admission to the catheter centre – during the index event	66,368 (87.2)	25,397 (83.1)	40,971 (89.8)	<0.01
coronarography- during the index event	63,517 (83.4)	23,863 (78.1)	39,654 (87)	<0.01
pain-to-first medical contact (minutes) – STEMI patients only	120 (55–300)	130 (60–341)	110 (50–280)	<0.01
first medical contact-to-balloon (minutes) -STEMI patients only	128 (88–202)	135 (92–213)	125 (85–196)	<0.01
total ischaemic time (minutes) – STEMI patients only	268 (170–495)	290 (185–540)	255 (165–470)	<0.01
pain-to-first medical contact (minutes) – NSTEMI patients only	206 (83–540)	215 (90–569)	200 (80–521)	0.13
first medical contact-to-balloon (minutes) - NSTEMI patients only	413 (191–823)	431 (197–868)	402 (190–789)	0.01
total ischaemic time (minutes) – NSTEMI patients only	582 (290–960)	600 (282–990)	575 (292–945)	0.09
angiography and PCI	52,316 (68.7)	19,091 (62.5)	33,225 (72.9)	<0.01
angiography and PCI, STEMI	28,094 (83)	10,127 (78.2)	17,967 (86)	<0.01
angiography and PCI, NSTEMI	24,222 (57.2)	8964 (50.9)	15,258 (61.8)	<0.01
only angiograpy, without PCI	11,021 (14.7)	4772 (15.6)	6429 (14.1)	<0.01
angiography and CABG	1843 (2.4)	555 (1.8)	1288 (2.8)	<0.01
angiography without PCI or CABG	9178 (12.3)	4217 (13.8)	5141 (11.3)	<0.01
MINOCA	3908 (5.1)	2093 (6.9)	1815 (4)	<0.01
CABG operation	1843 (2.4)	555 (1.8)	1288 (2.8)	<0.01
bleeding event during index event	1019 (1.3)	511 (1.7)	508 (1.1)	<0.01
lethal bleeding during index event	110 (0.1)	58 (0.2)	52 (0.1)	<0.01
CPR – during the index event	3939 (5.2)	1750 (5.7)	2189 (4.8)	<0.01
shock during treatment	4835 (6.3)	2219 (7.3)	2616 (5.7)	<0.01
mechanical ventilation	5903 (7.8)	2393 (7.8)	3510 (7.7)	<0.01
mechanical complications related to infarction	701 (0.9)	334 (1.1)	367 (0.8)	<0.01

**Outcomes**				
cardiac rehabilitation	14,355 (18.9)	5524 (18.1)	8831 (19.4)	<0.01
cardiac rehabilitation – early survivors (after 30 days of index event)	14,240 (21.2)	5448 (20.9)	8792 (21.4)	0.18
recurrent ACS episode	6749 (8.9)	2594 (8.5)	4155 (9.1)	<0.01
30-days mortality	9248 (12.1)	4522 (14.8)	4726 (10.4)	<0.01
1-year mortality	16,525 (21.7)	7964 (26.1)	8561 (18.6)	<0.01
2-years mortality	20,380 (26.8)	9617 (31.5)	10,763 (23.6)	<0.01
3-years mortality	23,660 (31.1)	11,047 (36.2)	12,613 (27.7)	<0.01
4-years mortality	26,185 (34.4)	12,122 (39.7)	14,063 (30.8)	<0.01
5-years mortality	28,147 (37)	12,950 (42.4)	15,197 (33.3)	<0.01

**Optimal drug therapy at discharge**				
ACEi/ARB	60,060 (78.9)	23,055 (75.5)	37,005 (81.1)	<0.01
Beta-receptor blocker	61,116 (80.3)	23,987 (78.5)	37,129 (81.4)	<0.01
ASA-therapy	64,665 (84.9)	24,850 (81.3)	24,850 (87.3)	<0.01
statin therapy	63,884 (83.9)	24,686 (80.8)	39,198 (86)	<0.01

**Abbreviations:** IQR: interquartile range, MWU: Mann-Whitney *U* test, CABG: coronary artery bypass graft, PCI: percutaneous coronary intervention, CPR: cardiopulmonary resuscitation, GFR: glomerular filtration rate, STEMI: ST-segment elevation myocardial infarction, NSTEMI: non-ST-segment elevation myocardial infarction, MINOCA: myocardial infarction with non-obstructive coronary artery disease, ACS: acute coronary syndrome, ACEi: angiotensin-converting enzyme inhibitor, ARB: angiotensin receptor blocker, ASA: acetylsalicylic acid.

**Table 2:** Factors predicting mortality in patients with acute coronary syndrome in Hungary (2014–2019). All-cause mortality was assessed as of March 25,2022, with an average follow-up period of 3.6 years.

	Binary logistic regression model	Cox-regression model		
parameter	adjusted hazard ratio of mortality (aHR, 95 % CI)	adjusted hazard ratio of 30-days-mortality (aHR, 95 % CI)	adjusted hazard ratio of 1-year-mortality (aHR, 95 % CI)	adjusted hazard ratio of 5-year-mortality (aHR, 95 % CI)
female sex	0.93 (0.89–0.97)	1.06 (1.01–1.11)	NS	0.97 (0.94–1.00)
age (/5 years of age increasing)	1.46 (1.44–1.47)	1.26 (1.24–1.27)	1.31 (1.30–1.32)	1.33 (1.32–1.34)
diabetes mellitus	1.46 (1.40–1.52)	1.16 (1.11–1.22)	1.22 (1.17–1.26)	1.28 (1.25–1.32)
serum creatitin level on admission (/10 mikromol/l increasing)	1.07 (1.06–1.07)	1.03 (1.03–1.03)	1.03 (1.03–1.03)	1.03 (1.03–1.03)
admission to the hospital with a catheter lab	0.78 (0.74–0.82)	0.90 (0.85–0.95)	0.89 (0.85–0.93)	0.88 (0.86–0.91)
STEMI diagnosis	1.06 (1.02–1.11)	1.55 (1.48–1.63)	1.31 (1.26–1.36)	1.13 (1.09–1.16)
cardiogenic shock on admission	1.52 (1.15–2.02)	1.34 (1.06–1.70)	1.30 (1.09–1.56)	1.35 (1.17–1.56)
cardiogenic shock during the index event	2.29 (2.01–2.60)	2.39 (2.21–2.59)	2.09 (1.95–2.23)	1.94 (1.83–2.06)
resuscitation during index event	3.42 (2.54–4.59)	2.15 (1.65–2.81)	2.60 (2.10–3.20)	2.79 (2.34–3.31)
PCI, if positive angiography findings	0.47 (0.44–0.49)	0.54 (0.51–0.57)	0.52 (0.50–0.55)	0.58 (0.56–0.60)
need of mechanical ventilation during index event	4.29 (3.87–4.74)	2.90 (2.70–3.11)	2.81 (2.64–2.98)	2.34 (2.22–2.47)
mechanical complication (VSR, tamponade, perforation)	1.84 (1.45–2.36)	1.47 (1.29–1.70)	1.57 (1.39–1.78)	1.67 (1.50–1.86)

**Abbreviations:** aHR: adjusted hazard ratio, CI: confidence interval, STEMI: ST-segment elevation myocardial infarction, PCI: percutaneous coronary intervention, VSR: ventricular septal rupture.

### 3.1 Sex differences in baseline characteristics and outcomes

#### 3.1.1 All patients

Differences in risk factors and presentation: The cardiovascular risk profile for female patients was less favorable when compared to their male counterparts. Upon presentation, females were seven years older than males, which may account for the higher prevalence of diabetes mellitus and hypertension observed among them. Active smoking was more common in men (30.2 % versus 19 % in women). A history of myocardial infarction was noted in 19.2 % of men versus 15.9 % of women, while 17.2 % of men had a history of prior percutaneous revascularization compared to 13 % of women. Additionally, prior surgical revascularization was reported in 5.6 % of men versus 3.4 % of women.

Upon admission, atrial fibrillation was notably more prevalent among women, with a rate of 10.3 %, compared to 8 % in men. Moreover, acute heart failure was more frequently observed in female patients, as 17.2 % presented with Killip stages II-IV at the time of admission, in contrast to 13.3 % of male patients. In comparison, 1.7 % of females and 1.4 % of males exhibited signs of cardiogenic shock. Additionally, the glomerular filtration rate (GFR) was lower in women, recorded at 62 ml/min/1.73m^2^, compared to 76 ml/min/1.73m^2^ for men.

NSTEMI was more prevalent among females, occurring in 57.6 % of cases compared to 54.6 % in males. Additionally, MINOCA was observed more frequently in women, with rates of 6.9 % versus 4 %.

Men had a greater likelihood of being directly admitted to a PCI-capable hospital, with rates of 80 % compared to 73.1 % for women. Furthermore, men were more often either directly admitted or subsequently transferred to a PCI-capable institution during their ACS event, with 89.8 % versus 83.1 % for women.

Differences in treatment: Coronary angiography was performed more frequently in men (87 % versus 78.1 %), and PCI was also more common (72.9 % compared to 62.5 %). For ST-segment elevation myocardial infarction (STEMI), the rates stood at 86 % for men versus 78.2 % for women, while for NSTEMI, the rates were 61.8 % for men compared to 50.9 % for women.

The ischemic time for women was more prolonged than for males (median patient onset of pain-to-first-medical-contact time was 110 min versus 130 min, median first-medical-contact-to-balloon time (a time recorded in Hungary that measures the time from first medical contact whatsoever (e.g., ambulance arrival) to the opening of the artery in the catherization laboratory. This time interval (different and longer than the „door to balloon time” commonly reported in the US) was 125 min versus 135 min, median total ischemic time was 255 min versus 290 min) in case of STEMI, first medical contact to-balloon time was longer in case of NSTEMI (402 min versus 431 min). Coronary artery bypass graft (CABG) operation was more frequent among males (1.8 % versus 2.8 %).

Women received less optimal (i.e., guideline-consistent) drug treatment at discharge than their male counterparts (angiotensin-converting enzyme inhibitor (ACEi)/ angiotensin receptor blocker (ARB) use 75.5 % versus 81.1 %, beta-blocker administration 78.5 % versus 81.4 %, acetylsalicylic acid (ASA) use 81.3 % versus 87.3 %, and statin drug administration 80.8 % versus 86 %). There were no sex differences in participation in the comprehensive cardiac rehabilitation program among early survivors (those alive after 30 days of the index event).

Differences in complications: There was a higher number of bleeding events after percutaneous coronary intervention among females, reported at 1.7 % compared to 1.1 % for males; lethal bleeding was more common (0.2 % vs. 0.1 %). The need for mechanical ventilation was more common (7.8 % and 7.7 %), and cardiogenic shock occurred more frequently during treatment (7.3 % versus 5.7 %).

Differences in outcomes: Women experienced higher unadjusted all-cause mortality rates compared to men: 30-day mortality was 14.8 % versus 10.4 %, 1-year mortality was 26.1 % versus 18.8 %, and 5-year mortality was 42.4 % versus 33.3 %.

With multivariable Cox-regression analysis, female sex was an independent determinant of death in the overall patient group: female sex was associated with increased mortality at 30 days (aHR 1.06 (1.01–1.11)) but was protective at 5 years (aHR: 0.97 (0.94–1.00)) (Table 2).

The influence of sex on the development of chronic heart failure remains unclear due to insufficient data. This highlights the need for further research to enhance our understanding in this area. Females were less likely to experience recurrent episodes of acute coronary syndrome during the follow-up period (8.5 % compared to 9.1 %) (Table 1, Table 2, Table 3).

**Table 3:** Mortality rates by sex of patients presenting with acute myocardial infarction in Hungary (2014–2019). Age-adjusted analysis with presentation of various subgroups.

			Without age-adjustment			Aged under 50 years			Aged 50–80 years			Aged over 80 years		
		all patients (%, N)	females (%, N)	males (%, N)	p-value (chi2 (Gupta et al., 2014))	females (%, N)	males (%, N)	p-value (chi2 (Gupta et al., 2014))	females (%, N)	males (%, N)	p-value (chi2 (Gupta et al., 2014))	females (%, N)	males (%, N)	p-value (chi2 (Gupta et al., 2014))
All patients	30 days	12.1 % (9248)	14.8 % (4522)	10.4 % (4726)	<0.01	2.2 % (42)	2.6 % (157)	0.28	10.4 % (2115)	9.2 % (3166)	<0.01	28.8 % (2365)	27.1 % (1403)	0.03
	1 year	21.7 % (16525)	26.1 % (7964)	18.8 % (8561)	<0.01	4.4 % (85)	4.3 % (258)	0.85	18.9 % (3852)	17.1 % (5873)	<0.01	49.1 % (4027)	47 % (2430)	0.02
	5 years	37 % (28147)	42.4 % (12950)	33.3 % (15197)	<0.01	8.2 % (160)	9 % (543)	0.31	33.1 % (6757)	31.6 % (10871)	<0.01	73.6 % (6033)	73.1 % (3783)	0.58
STEMI	30 days	12.8 % (4333)	16.9 % (2187)	10.3 % (2146)	<0.01	3.1 % (33)	2.9 % (112)	0.85	12.3 % (1094)	9.9 % (1540)	<0.01	35.7 % (1060)	31.7 % (494)	0.01
	1 year	19.6 % (6641)	25.3 % (3275)	16.1 % (3366)	<0.01	5.6 % (61)	4.6 % (176)	0.17	19 % (1690)	15.7 % (2444)	<0.01	51.4 % (1524)	47.9 % (746)	0.03
	5 years	31.4 % (10627)	38.3 % (4964)	27.1 % (5663)	<0.01	9 % (97)	8.9 % (340)	0.96	30.5 % (2712)	27.3 % (4237)	<0.01	72.6 % (2155)	69.8 % (1086)	0.04
NSTEMI	30 days	11.6 % (4915)	13.3 % (2335)	10.4 % (2580)	<0.01	1 % (9)	2 % (45)	0.06	8.9 % (1021)	8.6 % (1626)	0.46	24.9 % (1305)	25.1 % (909)	0.83
	1 year	23.4 % (9884)	26.6 % (4689)	21 % (5195)	<0.01	2.8 % (24)	3.7 % (82)	0.22	18.8 % (2162)	18.2 % (3429)	0.19	47.8 % (2503)	46.5 % (1684)	0.24
	5 years	41.4 % (17520)	45.4 % (7986)	38.6 % (9534)	<0.01	7.3 % (63)	9.1 % (203)	0.11	35.2 % (4045)	35.2 % (6634)	0.96	74.1 % (3878)	74.5 % (2697)	0.62
diabetics	30 days	14.8 % (3809)	17.1 % (1900)	13 % (1909)	<0.01	4.6 % (17)	3.6 % (37)	0.40	13 % (1016)	11.5 % (1369)	<0.01	30.1 % (867)	29 % (503)	0.04
	1 year	27 % (6955)	30.8 % (3416)	24.1 % (3539)	<0.01	7 % (26)	6.6 % (68)	0.80	24.3 % (1907)	21.8 % (2603)	<0.01	51.5 % (1483)	50.1 % (868)	0.37
	5 years	46 % (11855)	51.1 % (5661)	42.1 % (6194)	<0.01	15.7 % (58)	13.7 % (140)	0.34	43.2 % (3383)	39.5 % (4718)	<0.01	77.1 % (2220)	77.1 % (1336)	0.95
non-diabetics	30 days	10.8 % (5439)	13.5 % (2622)	9.1 % (2817)	<0.01	1.6 % (25)	2.4 % (120)	0.06	8.7 % (1099)	8 % (1797)	0.02	28.2 % (1498)	26.1 % (900)	0.04
	1 year	19 % (9570)	23.4 % (4548)	16.3 % (5022)	<0.01	3.7 % (59)	3.8 % (190)	0.95	15.5 % (1945)	14.6 % (3270)	0.02	47.8 % (2544)	45.4 % (1562)	0.03
	5 years	32.3 % (19292)	37.4 % (7289)	29.1 % (9003)	<0.01	6.5 % (102)	8 % (403)	0.40	26.8 % (3374)	27.4 % (6153)	0.24	71.1 % (2447)	71.7 % (3813)	0.57
PCI	30 days	7.9 % (4125)	9.6 % (1833)	6.9 % (2292)	<0.01	1.9 % (27)	2.4 % (119)	0.28	7.5 % (1054)	6.6 % (1675)	<0.01	20.8 % (752)	18.6 % (498)	0.03
	1 year	14.5 % (7570)	17.2 % (3269)	13 % (4301)	<0.01	4.1 % (58)	3.8 % (189)	0.61	13.8 % (1936)	12.5 % (3177)	<0.01	35.3 % (1275)	34.9 % (935)	0.76
	5 years	28.1 % (14661)	31.9 % (6088)	25.9 % (8573)	<0.01	7.7 % (110)	8.1 % (405)	0.66	26.7 % (3745)	25.5 % (6485)	0.01	61.8 % (2233)	62.8 % (1683)	0.39
non-PCI	30 days	10.3 % (1146)	10.7 % (507)	10.1 % (639)	0.28	0.6 % (2)	2.3 % (16)	0.05	9.1 % (303)	9.7 % (479)	0.36	19.5 % (202)	20.3 % (144)	0.65
	1 year	22.2 % (2463)	22.5 % (1067)	22 % (1396)	0.51	1.4 % (5)	4.5 % (31)	0.01	19.8 % (660)	21.1 % (1044)	0.15	38.7 % (402)	45.3 % (321)	0.01
	5 years	40.4 % (4477)	40.1 % (1897)	40.7 % (2580)	0.52	5.5 % (20)	10 % (69)	0.01	35.5 % (1185)	40.1 % (1986)	<0.01	66.7 % (692)	74.2 % (525)	<0.01

**Abbreviations:** STEMI: ST-segment myocardial infarction, NSTEMI: non-ST-segment elevation myocardial infarction, PCI: percutaneous coronary intervention.

### 3.2 Age-adjusted analysis

**Fig. 1:**
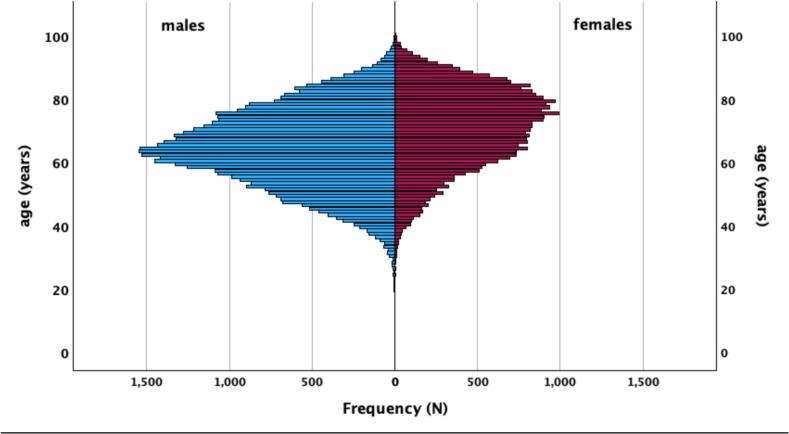
- Age and sex distribution of all patients presenting with acute myocardial infarction in Hungary between 2014 and 2019.

Age is a well-established and unmodifiable risk factor for cardiovascular disease. Upon admission, females were older, with a median age difference of 7.3 years (Table 1). This discrepancy is further illustrated in the age distribution graph (Fig. 1). In Hungary, the overall life expectancy is 74.4 years, with females averaging 77.8 years and males 70.9 years (World health Organization, 2025). The older age of women at the time of presentation may adversely affect their prognosis and survival rates; however, their longer lifespan could positively influence their outcomes. For a more detailed analysis, the patients were grouped by their age.

### 3.3 Patients under 50 years

Diabetes mellitus and active smoking were found to be more prevalent among females, who also presented with more advanced circulatory failure and elevated creatinine levels upon admission, suggesting poorer renal function despite the limitations of this indicator. The incidence of NSTEMI and MINOCA was notably higher in this group. Overall, the patterns of revascularization were less favorable for females. Furthermore, there was a greater incidence of shock during hospitalization among females, and they were less likely to participate in rehabilitation programs. The use of ACEi or ARB was also less common, potentially due to concerns regarding childbearing potential.

Multivariable statistical analysis indicated that diabetes mellitus, impaired renal function, the occurrence of shock during care, and the requirement for mechanical ventilation are key predictors of adverse outcomes associated with all-cause mortality, both in the mid- and long-term. Furthermore, PCI emerged as a protective factor in this context (Supplementary Table 4).

### 3.4 Patients over 80 years

Females tended to be older and showed a higher prevalence of diabetes mellitus and hypertension. In contrast, males were more likely to present with hyperlipidemia, active smoking, and a history of cardiovascular issues. Upon admission, females had a higher rate of heart failure, while males demonstrated worse renal function and had a higher incidence of prehospital cardiac arrest. Although STEMI was more prevalent among females, their revascularization treatment remained less favorable compared to their male counterparts. Moreover, the need for mechanical ventilation was more frequently observed in males, and recurrent ACS events were also more common in this group. Unadjusted mortality rates indicated that females experienced worse short- and mid-term survival outcomes, while pharmacological therapy at discharge was more optimal for males. A multivariable statistical analysis indicated that diabetes mellitus, atrial fibrillation, impaired renal function, STEMI diagnosis, shock, and mechanical ventilation are significant predictors of higher all-cause mortality. Conversely, PCI was shown to provide a protective effect for mid- and long-term outcomes. An additional predictor of worse long-term outcomes was prehospital cardiac arrest (Supplementary Table 5).

### 3.5 Mortality, subgroup analysis

There was no significant difference in mortality rates for patients under 50 years of age; however, among those who did not undergo PCI, males had poorer outcomes regarding all-cause mortality.

For patients aged 50 to 80, females had worse outcomes in terms of short-term, mid-term, and long-term mortality. In cases of NSTEMI, no mortality differences were observed. Among nondiabetic patients, long-term mortality rates were comparable between sexes. Nevertheless, males who did not receive PCI had a notably worse long-term prognosis.

## 4 Discussion

This study provides a comprehensive analysis of sex differences in the prognosis and management of ACS, showing significant differences in treatment modality chosen and treatment outcomes between male and female patients.

### 4.1 Sex differences

Female patients exhibited less favorable cardiovascular risk profiles, as demonstrated by higher rates of diabetes and hypertension, and most importantly, older age. Recent data reveal an increased incidence of diabetes mellitus in women, which contradicts the findings of previous studies (*Lancet*, n.d.). This inconsistency highlights the need for further investigation into the underlying factors contributing to this unexpected outcome.

Females were seven years older at presentation, faced less favorable treatment times, and were less likely to receive revascularization. This may be explained by their higher rates of MINOCA and NSTEMI among them. Additionally, female patients experienced higher rates of complications during treatment and demonstrated lower adherence to guideline-consistent medication therapy at discharge. In contrast, male patients were more likely to receive timely interventions and to undergo PCI. However, among those who did not receive PCI, males exhibited poorer long-term mortality outcomes.

The study highlights significant sex associated differences in mortality outcomes for ACS patients (Table 1). While at first sight, healthcare provider bias towards women may seem an easy explanation, the noted mortality differences are likely multifactorial and cannot be pinned to any one single factor. Interestingly, while unadjusted mortality rates were higher in women, multivariable analysis indicated a mortality benefit for females at five years. As stated earlier, the longer life expectancy of women and their older age at the time of presentation need to be considered. Therefore, sex differences, treatment quality, and comorbidities all may influence the observed disparities rather than inherent biological differences or provider bias alone (Fig. 1).

### 4.2 Age-adjustment

Diabetes mellitus is a significant predictor of increased all-cause mortality, regardless of age.

When considering short-term survival, patients with STEMI typically have poorer outcomes, whereas those with NSTEMI tend to show worse prognoses in the mid- and long-term. However, this pattern does not apply to patients under 50 years of age, as there is no significant difference in long-term prognosis between NSTEMI and STEMI in this younger group (5-year mortality χ^2^ *p* = 0.57).

Percutaneous coronary intervention provides a survival benefit even for individuals over the age of 80. There is an urgent need for timely revascularization strategies for patients experiencing STEMI. Healthcare institutions should focus on minimizing treatment delays to improve patient outcomes. Patients aged over 80, particularly females, tend to demonstrate a less favorable prognosis in both the short and mid-term. Notably, in the case of NSTEMI, no significant difference was observed in mortality rates.

The study also emphasizes that older patients, particularly those over 80, present unique challenges. The higher prevalence of comorbidities in this age group requires a more nuanced understanding of their treatment needs and the potential barriers to achieving successful outcomes.

The higher prevalence of NSTEMI among younger women, coupled with their longer ischemic times and reduced likelihood of receiving timely PCI, further complicates their clinical outcomes and emphasizes the need for improved awareness and education regarding ACS symptoms in this demographic. In individuals aged 80 and older, there is a notable trend indicating that a higher percentage of females are diagnosed with STEMI compared to their male counterparts. Despite this increased prevalence, it is concerning that the revascularization rate for these female patients remains significantly lower than that of males. This disparity highlights the need for further investigation into factors contributing to differences in treatment and outcomes based on sex in this age group (Supplementary Table 4).

Among diabetic patients, a short-term disparity is evident, with females experiencing a poorer prognosis, similar to the outcomes reported in non-diabetic individuals within both the short and mid-term periods.

Regardless of age, the presence of cardiogenic shock upon admission or its subsequent development is a significant predictor of poorer outcomes. This highlights the critical importance of early detection (Society for Cardiovascular Angiography & Interventions Shock Stage A and B) and timely intervention, including early revascularization, in managing circulatory failure. The need for mechanical ventilation is associated with a significantly less favorable prognosis.

### 4.3 Limitations

The analysis conducted in this study was retrospective. It was necessary to implement specific statistical adjustments to account for missing data, including anthropometric measurements, smoking status, treatment durations, and adherence to secondary prevention medication. Furthermore, the socioeconomic backgrounds of the patient population examined remain unknown. There is also a lack of information regarding female-specific cardiovascular risk factors, such as menopausal status, eclampsia, and migraine. This analysis is based on data from Hungarian patients, whose cardiovascular risk profiles are generally perceived to be less favorable than those of their counterparts in Western Europe or the United States (Blöndal et al., 2022; Hellgren et al., 2022). It should be noted that only all-cause mortality data are available for this study.

## 5 Conclusion

The findings of this study reveal significant sex differences in the management and outcomes of patients with acute coronary syndrome. The elevated mortality rates observed among female patients can be attributed to a combination of factors, including older age, higher rates of comorbidities, and disparities in treatment approaches. Nonetheless, further research is essential. Recognizing the multifaceted nature of these differences is critical for developing effective, gender-sensitive treatment strategies that can enhance outcomes for all acute coronary syndrome patients.

## CRediT authorship contribution statement

**Csaba Sári:** Writing – review & editing, Writing – original draft, Validation, Methodology, Formal analysis, Data curation, Conceptualization. **Christian M. Heesch:** Writing – review & editing, Writing – original draft, Methodology. **Attila János Kovács:** Writing – review & editing. **Péter Andréka:** Writing – review & editing, Writing – original draft, Conceptualization.

## Sources of funding

Nothing to declare.

## Declaration of competing interest

The authors declare that they have no known competing financial interests or personal relationships that could have appeared to influence the work reported in this paper.

## Data Availability

The data that has been used is confidential.

## Appendix A Supplementary data

Supplementary Tables - Sex distribution and mortality predictors in myocardial infarction patients under 50 years and over 80 years old in Hungary (2014-2019). Image 1

## References

[bb0005] Akhter N., Milford-Beland S., Roe M.T. (2009). Gender differences among patients with acute coronary syndromes undergoing percutaneous coronary intervention in the American College of Cardiology-National Cardiovascular Data Registry (ACC-NCDR). Am. Heart J..

[bb0010] Berthillot C., Stephan D., Chauvin M., Roul G. (2010). In-hospital complications after invasive strategy for the management of non STEMI: women fare as well as men. BMC Cardiovasc. Disord..

[bb0015] Blöndal M., Ainla T., Eha J. (2022). Comparison of management and outcomes of ST-segment elevation myocardial infarction patients in Estonia, Hungary, Norway, and Sweden according to national ongoing registries. Eur Heart J Qual Care Clin Outcomes.

[bb0020] Byrne R.A., Rossello X., Coughlan J.J. (2023). 2023 ESC guidelines for the management of acute coronary syndromes. Eur. Heart J..

[bb0025] Canto J.G., Goldberg R.J., Hand M.M. (2007). Symptom presentation of women with acute coronary syndromes: myth vs reality. Arch. Intern. Med..

[bb0030] Dey S., Flather M.D., Devlin G. (2009). Sex-related differences in the presentation, treatment and outcomes among patients with acute coronary syndromes: the global registry of acute coronary events. Heart.

[bb0035] Du X., Patel A., Li X. (2017). Treatment and outcomes of acute coronary syndromes in women: an analysis of a multicenter quality improvement Chinese study. Int. J. Cardiol..

[bb0040] Farb A., Burke A.P., Tang A.L. (1996). Coronary plaque erosion without rupture into a lipid core. A frequent cause of coronary thrombosis in sudden coronary death. Circulation.

[bb0045] Gebhard C.E., Gebhard C., Maafi F. (2019). Impact of summer season on pre-hospital time delays in women and men undergoing primary percutaneous coronary intervention. Sci. Total Environ..

[bb0050] Gupta A., Wang Y., Spertus J.A. (2014). Trends in acute myocardial infarction in young patients and differences by sex and race, 2001 to 2010. J. Am. Coll. Cardiol..

[bb0055] Hao K., Takahashi J., Ito K. (2015). Clinical characteristics of patients with acute myocardial infarction who did not undergo primary percutaneous coronary intervention- report from the MIYAGI-AMI registry study. Circ. J..

[bb0060] Hao Y., Liu J., Liu J. (2019). Sex differences in in-hospital management and outcomes of patients with acute coronary syndrome. Circulation.

[bb0065] Hellgren T., Blöndal M., Jortveit J. (2022). Sex-related differences in the management and outcomes of patients hospitalized with ST-elevation myocardial infarction: a comparison within four European myocardial infarction registries. Eur Heart J Open.

[bb0070] Huber E., Le Pogam M.A., Clair C. (2022). Sex related inequalities in the management and prognosis of acute coronary syndrome in Switzerland: cross sectional study. BMJ Med.

[bb0075] Khan E., Brieger D., Amerena J. (2018). Differences in management and outcomes for men and women with ST-elevation myocardial infarction. Med. J. Aust..

[bb0080] Kuehnemund L., Koeppe J., Feld J. (2021). Gender differences in acute myocardial infarction-a nationwide German real-life analysis from 2014 to 2017. Clin. Cardiol..

[bb0085] Kunadian V., Mossop H., Shields C. (2024). Invasive treatment strategy for older patients with myocardial infarction. N. Engl. J. Med..

[bb0125] World health Organization (2025).

[bb0090] Collaboration NCDRF. Worldwide trends in diabetes prevalence and treatment from 1990 To 2022: a pooled analysis of 1108 population-representative studies with 141 million participants. Lancet 2024;404:2077–2093. doi: 10.1016/S0140-6736(24)02317-1. PMC7616842 39549716

[bb0095] Lawton J.S., Tamis-Holland J.E., Bangalore S. (2022). 2021 ACC/AHA/SCAI guideline for coronary artery revascularization: executive summary: a report of the American College of Cardiology/American Heart Association joint committee on clinical practice guidelines. J. Am. Coll. Cardiol..

[bb0100] Lichtman J.H., Leifheit E.C., Safdar B. (2018). Sex differences in the presentation and perception of symptoms among young patients with myocardial infarction: evidence from the VIRGO study (variation in recovery: role of gender on outcomes of young AMI patients). Circulation.

[bb0105] Lunova T., Komorovsky R., Klishch I. (2023). Gender differences in treatment delays, management and mortality among patients with acute coronary syndrome: a systematic review and Meta-analysis. Curr. Cardiol. Rev..

[bb0110] Martin S.S., Aday A.W., Allen N.B. (2025). 2025 heart disease and stroke statistics: a report of US and global data from the American Heart Association. Circulation.

[bb0115] Mehta L.S., Beckie T.M., DeVon H.A. (2016). Acute myocardial infarction in women: a scientific statement from the American Heart Association. Circulation.

[bb0120] Meyer M.R., Bernheim A.M., Kurz D.J. (2019). Gender differences in patient and system delay for primary percutaneous coronary intervention: current trends in a Swiss ST-segment elevation myocardial infarction population. Eur. Heart J. Acute Cardiovasc. Care.

[bb0130] Redfors B., Angerås O., Råmunddal T. (2015). Trends in gender differences in cardiac care and outcome after acute myocardial infarction in Western Sweden: a report from the Swedish web system for enhancement of evidence-based Care in Heart Disease Evaluated According to recommended therapies (SWEDEHEART). J. Am. Heart Assoc..

[bb0135] Resurrección D.M., Moreno-Peral P., Gómez-Herranz M. (2019). Factors associated with non-participation in and dropout from cardiac rehabilitation programmes: a systematic review of prospective cohort studies. Eur. J. Cardiovasc. Nurs..

[bb0140] Roswell R.O., Kunkes J., Chen A.Y. (2017). Impact of sex and contact-to-device time on clinical outcomes in acute ST-segment elevation myocardial infarction-findings from the National Cardiovascular Data Registry. J. Am. Heart Assoc..

[bb0145] Scott P.E., Unger E.F., Jenkins M.R. (2018). Participation of women in clinical trials supporting FDA approval of cardiovascular drugs. J. Am. Coll. Cardiol..

[bb0150] Selzer A., Langston M., Ruggeroli C., Cohn K. (1976). Clinical syndrome of variant angina with normal coronary arteriogram. N. Engl. J. Med..

[bb0155] Smilowitz N.R., Mahajan A.M., Roe M.T. (2017). Mortality of myocardial infarction by sex, age, and obstructive coronary artery disease status in the ACTION registry-GWTG (acute coronary treatment and intervention outcomes network registry-get with the guidelines). Circ. Cardiovasc. Qual. Outcomes.

[bb0160] Thompson E.A., Ferraris S., Gress T., Ferraris V. (2005). Gender differences and predictors of mortality in spontaneous coronary artery dissection: a review of reported cases. J. Invasive Cardiol..

[bb0165] Timmis A., Townsend N., Gale C.P. (2020). European Society of Cardiology: cardiovascular disease statistics 2019. Eur. Heart J..

