# Supplementary material for: Sex-related differences in care and prognosis in acute coronary syndrome
Source: Prev Med Rep. 2025 Jun 7;55:103131. doi: 10.1016/j.pmedr.2025.103131 (PMC12178922; doi:10.1016/j.pmedr.2025.103131)
Supplement: Supplementary file 1 — Supplementary Tables - Sex distribution and mortality predictors in myocardial infarction patients under 50 years and over 80 years old in Hungary (2014-2019). [file mmc1.docx]

***Supplementary Table 4***

**Sex distribution and mortality predictors in myocardial infarction patients under 50 years old in Hungary (2014-2019)**

| sex differences | | | | multivariable analysis | |
| --- | --- | --- | --- | --- | --- |
|  | females, number of cases (%), or median and IQR | males, number of cases (%), or median and IQR | p-value (chi2, T-test, MWU) | adjusted hazard ratio  1-year mortality | adjusted hazard ratio  5-years mortality |
| Sex distribution | 1945 (24.3) | 6046 (75.7) |  |  |  |
| **Risk factors** | | | | | |
| Age (years) | 45 (42-48) | 45 (42-48) | 0.60 |  |  |
| Diabetes mellitus | 370 (19) | 1025 (17) | 0.04 | 1.79 (1.40-2.29) | 1.91 (1.61-2.27) |
| Hypertension | 1105 (56.8) | 3363 (55.6) | 0.45 |  |  |
| Hyperlipidaemia | 1406 (23.3) | 424 (21.8) | 0.37 |  |  |
| Anamnestic myocardial infarction | 156 (8) | 559 (9.2) | 0.26 |  |  |
| Anamnestic CABG | 17 (0.9) | 52 (0.9) | 0.35 |  |  |
| Anamnestic PCI | 153 (7.9) | 534 (8.8) | 0.38 |  |  |
| Active smoking | 3496 (57.8) | 1088 (55.9) | <0.01 |  |  |
| **Admission** | | | | | |
| Prehospital CPR | 73 (3.8) | 231 (3.8) | 0.19 |  | 2.92 (1.05-8.14) |
| Atrial fibrillation | 19 (1) | 66 (1.1) | 0.87 |  |  |
| Killip I | 1751 (90) | 5566 (92.1) | 0.02 |  |  |
| Killip II | 122 (6.3) | 334 (5.5) |  |  |  |
| Killip III | 34 (1.7) | 75 (1.2) |  |  |  |
| Killip IV | 20 (1) | 43 (0.7) |  |  |  |
| Glomerular flitration rate (ml/min/1,73m^2^) | 99 (84-107) | 98 (81-108) | 0.71 |  |  |
| Serum creatinine (umol/l) | 78 (64-90) | 61 (47-74) | <0.01 | 1.28 (1.01-1.63) | 1.22 (1.04-1.45) |
| **ACS characteristics, treatment** | | | | | |
| STEMI diagnosis | 1080 (55.5) | 3808 (63) | <0.01 |  |  |
| NSTEMI diagnosis | 865 (44.5) | 2238 (37) | <0.01 |  |  |
| Coronary angiography | 1790 (92) | 5711 (94.5) | <0.01 |  |  |
| PCI, if positive angiography findings | 1424 (79.7) | 5007 (87.9) | <0.01 | 0.47 (0.36-0.61) | 0.58 (0.49-0.70) |
| Angiography and PCI - STEMI | 936 (90.9) | 3412 (93) | 0.02 |  |  |
| Angiography and PCI - NSTEMI | 488 (64.6) | 1595 (78.7) | <0.01 |  |  |
| MINOCA | 229 (11.8) | 380 (6.3) | <0.01 |  |  |
| CABG operation | 101 (0.5) | 70 (1.2) | <0.01 |  |  |
| Bleeding event after PCI | 9 (0.5) | 20 (0.3) | 0.62 |  |  |
| Lethal bleeding event after PCI | 1 (0.1) | 3 (0.1) | 0.89 |  |  |
| CPR – during the index event | 64 (3.3) | 158 (2.6) | 0.08 |  |  |
| Shock during treatment | 67 (3.4) | 147 (2.4) | 0.02 | 3.24 (2.35-4.45) | 3.19 (2.40-4.24) |
| Mechanical ventilation | 97 (5) | 258 (4.3) | 0.18 | 13.52 (9.50-19.24) | 5.94 (4.43-7.97) |
| Mechanical complications related to infarction | 7 (0.4) | 36 (0.6%) | 0.22 |  |  |
| **Outcomes** | | | | | |
| Cardiac rehabilitation | 419 (21.5) | 1434 (23.7) | 0.05 |  |  |
| Cardiac rehabilitation – early survivors (after 30 days of index event) | 419 (22) | 1430 (24.3) | 0.04 |  |  |
| Recurrent ACS episode | 146 (7.5) | 506 (8.4) | 0.23 |  |  |
| 30-days mortality | 42 (2.2) | 157 (2.6) | 0.28 |  |  |
| 1-year mortality | 85 (4.4) | 258 (4.3) | 0.85 |  |  |
| 2-years mortality | 111 (5.7) | 341 (5.6) | 0.91 |  |  |
| 3-years mortality | 132 (6.8) | 417 (6.9) | 0.87 |  |  |
| 4-years mortality | 144 (7.4) | 482 (8) | 0.42 |  |  |
| 5-years mortality | 160 (8.2) | 543 (9) | 0.31 |  |  |
| **Optimal drug therapy at discharge** | | | | | |
| ACEi/ARB | 1557 (80.1) | 5125 (84.8) | <0.01 |  |  |
| Beta-receptor blocker | 1644 (84.5) | 5195 (85.9) | 0.16 |  |  |
| ASA-therapy | 1814 (93.3) | 5658 (93.6) | 0.62 |  |  |
| Statin therapy | 1735 (89.2) | 5491 (90.8) | 0.05 |  |  |

**Abbreviations:** IQR: interquartile range, MWU: Mann-Whitney U test, CABG: coronary artery bypass graft, PCI: percutaneous coronary intervention, CPR: cardiopulmonary resuscitation, GFR: glomerular filtration rate, STEMI: ST-segment elevation myocardial infarction, NSTEMI: non-ST-segment elevation myocardial infarction, MINOCA: myocardial infarction with non-obstructive coronary artery disease, ACS: acute coronary syndrome, ACEi: angiotensin-converting enzyme inhibitor, ARB: angiotensin receptor blocker, ASA: acetylsalicylic acid

***Supplementary Table 5***

**Sex distribution and mortality predictors in myocardial infarction patients over 80 years old in Hungary (2014-2019)**

| sex differences | | | | multivariable analysis | |
| --- | --- | --- | --- | --- | --- |
|  | females, number of cases (%), or median and IQR | males, number of cases (%), or median and IQR | p-value (chi2, T-test, MWU) | adjusted hazard ratio  1-year mortality | adjusted hazard ratio  5-years mortality |
| Sex distribution | 8202 (61.3) | 5187 (38.7) |  |  |  |
| **Risk factors** | | | | | |
| Age (years) | 85 (82-88) | 84 (82-87) | <0.01 |  |  |
| Diabetes mellitus | 2881 (35.1) | 1732 (33.5) | 0.05 | 1.10 (1.05-1.16) | 1.14 (1.10-1.19) |
| Hypertension | 7362 (89.8) | 4459 (86.2) | <0.01 |  |  |
| Hyperlipidaemia | 2192 (26.7) | 1482 (28.6) | <0.01 |  |  |
| Anamnestic myocardial infarction | 1572 (19.2) | 1391 (26.9) | <0.01 |  |  |
| Anamnestic CABG | 276 (3.4) | 457 (8.8) | <0.01 |  |  |
| Anamnestic PCI | 999 (12.2) | 1031 (19.9) | <0.01 |  |  |
| Active smoking | 145 (1.8) | 231 (4.5) | <0.01 |  |  |
| **Admission** | | | | | |
| Prehospital CPR | 159 (1.9) | 129 (2.5) | 0.02 |  | 1.31 (1.03-1.69) |
| Atrial fibrillation | 1658 (20.2) | 1003 (19.4) | 0.13 | 1.35 (1.16-1.57) | 1.40 (1.24-1.58) |
| Killip I | 5877 (71.7) | 3876 (74.9) | <0.01 |  |  |
| Killip II | 1575 (19.2) | 847 (16.4) |  |  |  |
| Killip III | 436 (5.3) | 276 (5.3) |  |  |  |
| Killip IV | 172 (2.1) | 111 (2.1) |  | 1.30 (1.05-1.61) |  |
| Glomerular flitration rate (ml/min/1,73m^2^) | 43 (30-60) | 49 (36-65) | <0.01 |  |  |
| Serum creatinine (umol/l) | 95 (71-128) | 111 (86-145) | <0.01 | 1.44 (1.36-1.52) | 1.37 (1.32-1.43) |
| **ACS characteristics, treatment** | | | | | |
| STEMI diagnosis | 2967 (36.2) | 1556 (30.1) | <0.01 | 1.40 (1.33-1.48) | 1.21 (1.16-1.27) |
| NSTEMI diagnosis | 5235 (63.8) | 3618 (69.9) | <0.01 |  |  |
| Coronary angiography | 4663 (56.9) | 3401 (65.7) | <0.01 |  |  |
| PCI, if positive angiography findings | 3514 (77.7) | 2678 (79.1) | <0.01 | 0.45 (0.43-0.47) | 0.51 (0.49-0.54) |
| Angiography and PCI - STEMI | 1824 (87.2) | 1104 (89.9) | 0.02 |  |  |
| Angiography and PCI - NSTEMI | 1790 (69.6) | 1574 (72.9) | 0.02 |  |  |
| MINOCA | 427 (5.2) | 226 (4.4) | <0.01 |  |  |
| CABG operation | 58 (0.7) | 60 (1.2) | <0.01 |  |  |
| Bleeding event after PCI | 183 (2.2) | 129 (2.5) | 0.55 |  |  |
| Lethal bleeding event after PCI | 24 (0.3) | 11 (0.2) | 0.61 |  |  |
| CPR – during the index event | 678 (8.3) | 460 (8.9) | 0.12 |  |  |
| Shock during treatment | 881 (10.7) | 542 (10.5) | 0.63 | 3.67 (3.36-4.00) | 3.28 (3.03-3.56) |
| Mechanical ventilation | 710 (8.7) | 521 (10.1) | <0.01 | 1.92 (1.75-2.11) | 1.68 (1.54-1.83) |
| Mechanical complications related to infarction | 120 (1.5) | 60 (1.2) | 0.14 |  |  |
| **Outcomes** | | | | | |
| Cardiac rehabilitation | 912 (11.1) | 561 (10.8) | 0.62 |  |  |
| Cardiac rehabilitation – early survivors (after 30 days of index event) | 873 (15) | 534 (14.2) | 0.28 |  |  |
| Recurrent ACS episode | 628 (7.7) | 467 (9) | <0.01 |  |  |
| 30-days mortality | 2365 (28.8) | 1403 (27.1) | 0.03 |  |  |
| 1-year mortality | 4027 (49.1) | 2430 (47) | 0.02 |  |  |
| 2-years mortality | 4735 (57.7) | 2921 (56.5) | 0.15 |  |  |
| 3-years mortality | 5314 (64.8) | 3325 (64.3) | 0.54 |  |  |
| 4-years mortality | 5729 (69.8) | 3581 (69.2) | 0.44 |  |  |
| 5-years mortality | 6033 (73.6) | 3783 (73.1) | 0.58 |  |  |
| **Optimal drug therapy at discharge** | | | | | |
| ACEi/ARB | 5227 (63.7) | 3451 (66.7) | <0.01 |  |  |
| Beta-receptor blocker | 5610 (68.4) | 3598 (69.5) | <0.01 |  |  |
| ASA-therapy | 5514 (67.1) | 3961 (71.3) | <0.01 |  |  |
| Statin therapy | 5575 (68) | 3679 (71.1) | <0.01 |  |  |

**Abbreviations:** IQR: interquartile range, MWU: Mann-Whitney U test, CABG: coronary artery bypass graft, PCI: percutaneous coronary intervention, CPR: cardiopulmonary resuscitation, GFR: glomerular filtration rate, STEMI: ST-segment elevation myocardial infarction, NSTEMI: non-ST-segment elevation myocardial infarction, MINOCA: myocardial infarction with non-obstructive coronary artery disease, ACS: acute coronary syndrome, ACEi: angiotensin-converting enzyme inhibitor, ARB: angiotensin receptor blocker, ASA: acetylsalicylic aci
